# A 12-channel flexible receiver coil for accelerated tongue imaging

**DOI:** 10.1007/s10334-019-00824-5

**Published:** 2020-01-16

**Authors:** Luuk Voskuilen, Paul de Heer, Lisette van der Molen, Alfons J. M. Balm, Ferdinand van der Heijden, Gustav J. Strijkers, Ludi E. Smeele, Aart J. Nederveen

**Affiliations:** 1 Department of Head and Neck Oncology and Surgery, Netherlands Cancer Institute, Antoni Van Leeuwenhoek Hospital, Plesmanlaan 121, 1066 CX Amsterdam, The Netherlands; 2 Department of Radiology and Nuclear Medicine, Amsterdam UMC, University of Amsterdam, Amsterdam, Netherlands; 3 Department of Oral and Maxillofacial Surgery, Academic Centre for Dentistry Amsterdam and Academic Medical Center, University of Amsterdam and VU University Amsterdam, Amsterdam, Netherlands; 4 Department of Oral and Maxillofacial Surgery, Amsterdam UMC, University of Amsterdam, Amsterdam, Netherlands; 5 Department of Robotics and Mechatronics, MIRA Institute, University of Twente, Enschede, Netherlands; 6 Biomedical Engineering and Physics, Amsterdam UMC, University of Amsterdam, Amsterdam, Netherlands

**Keywords:** Tongue, Acceleration, Deglutition, Diffusion tensor imaging, Magnetic resonance imaging, Cine

## Abstract

**Objective:**

MRI of the tongue requires acceleration to minimise motion artefacts and to facilitate real-time imaging of swallowing. To accelerate tongue MRI, we designed a dedicated flexible receiver coil.

**Materials and methods:**

We designed a flexible 12-channel receiver coil for tongue MRI at 3T and compared it to a conventional head-and-neck coil regarding SNR and g-factor. Furthermore, two accelerated imaging techniques were evaluated using both coils: multiband (MB) diffusion-tensor imaging (DTI) and real-time MRI of swallowing.

**Results:**

The flexible coil had significantly higher SNR in the anterior (2.1 times higher, *P* = 0.002) and posterior (2.0 times higher, *P* < 0.001) parts of the tongue, while the g-factor was lower at higher acceleration. Unlike for the flexible coil, the apparent diffusion coefficient (*P* = 0.001) and fractional anisotropy (*P* = 0.008) deteriorated significantly while using the conventional coil after accelerating DTI with MB. The image quality of real-time MRI of swallowing was significantly better for hyoid elevation (*P* = 0.029) using the flexible coil.

**Conclusion:**

Facilitated by higher SNR and lower g-factor values, our flexible tongue coil allows faster imaging, which was successfully demonstrated in MB DTI and real-time MRI of swallowing.

**Electronic supplementary material:**

The online version of this article (10.1007/s10334-019-00824-5) contains supplementary material, which is available to authorized users.

## Introduction

Accelerated imaging has been an intensively researched field in MRI over the past two decades, which resulted in techniques such as parallel imaging [1, 2], multiband imaging [3, 4], and compressed sensing [5]. As acceleration reduces the total scan time, the incidence of motion artefacts decreases. In approximately 20% of all MRI examinations, an imaging sequence has to be repeated due to motion artefacts [6]. Not only have these artefacts been estimated to cost the hospital $115,000 per scanner per year [6], they may also prevent a radiologist from determining the correct diagnosis.

MRI of the tongue may especially benefit from acceleration, as it is prone to motion artefacts due to breathing and swallowing. Besides motion artefact reduction, acceleration techniques have recently been used to develop new imaging protocols, such as real-time dynamic imaging of speech [7] and swallowing [8]. Additionally, acceleration may improve the clinical feasibility of imaging techniques that currently take too much time for routine clinical examinations, such as high-angular resolution diffusion imaging of the tongue [9].

In general, acceleration reduces the signal-to-noise ratio (SNR). In parallel imaging, SNR loss is related to the square root of the acceleration factor and a geometry-specific noise-amplification factor (g-factor). In the recently developed multiband (MB) imaging approach, several slices are acquired simultaneously. As the number of k-space samples acquired is not decreased in MB imaging, the SNR loss due to acceleration is no longer dependent on the square root of the acceleration factor, but only on the g-factor [4]. In another acceleration technique, compressed sensing, the relationship between acceleration and SNR loss is not as straight-forward, as compressed sensing has an inherent denoising effect [5]. Nevertheless, improving SNR has been attributed to milder artefacts and thus better image quality in real-time imaging [10]. In summary, in order to maintain image quality after acceleration in tongue MRI, we hypothesize that the SNR should be increased and the g-factor reduced.

The SNR and g-factor can both be improved by designing a receiver coil specifically for the tongue, as the SNR can be increased by using small surface-coil elements that have an intrinsically higher SNR, and the g-factor can be reduced by increasing the number of coil elements that are sensitive to the anatomy of interest. Consequently, such a custom coil allows for higher acceleration than conventional coils, as has been proven for the breast [11], heart [12], and upper airway at 1.5T [10] and 3T [13].

These coil designs include either a rigid housing [10–12] or a rigid support structure [13] to improve the robustness of the coil. As coil are often designed to be used with a variety of subjects, a rigid coil may therefore prevent the coil elements from being placed as close as possible to the subject, which decreases the maximal SNR that could be achieved. Additionally, these rigid coils may restrict a subject’s mobility or the delivery of a contrast agent, which makes the study of swallowing of MRI more difficult. A flexible array of small coil elements may therefore not only allow the subject to move more freely, but it also improves SNR.

In this study, we designed a flexible receiver coil with 12 coil elements to accelerate tongue MRI at 3T. We compared the flexible coil to a conventional 16-channel neurovascular coil for SNR and g-factor characteristics. Furthermore, the two coils were compared on two applications: Diffusion-weighted multiband imaging of the tongue, and real-time MRI of swallowing using compressed sensing.

## Materials and methods

### Volunteers

We included five healthy volunteers (2 female; mean age 27 years, range 23–29 years), who gave written informed consent, according to the regulations of our institution. Volunteers were excluded if they had orthodontic appliances or any contraindications to MRI. The volunteers were scanned in a 3T Philips Ingenia scanner (Philips Healthcare, Best, Netherlands). Using identical scanning protocols, we scanned the volunteers first with the conventional neurovascular coil, and afterwards with the flexible coil.

### Flexible coil design

**Fig. 1:**
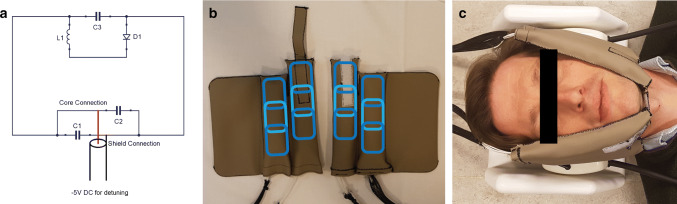
Design and lay-out of the flexible coil: **a** the schematic of a single coil element with three capacitors (C1–3), one inductor (L1), and one diode (D1). **b** Three of these elements were assembled in a phased array indicated by the blue rounded rectangles. **c** The coil was placed directly on the cheeks of a subject. The synthetic leather strap and flaps, which were placed between the headphones and the subject’s head, kept the coil in position. The white 3D-printed headrest minimised head rotation during scanning

Two separate coil compartments were created with six flexible copper coil elements each. The elements (53 by 32 mm) were printed on a flexible circuit board, on which components were soldered according to the design displayed in Fig. 1a (MRcoils, Zaltbommel, Netherlands). Each element has a small detune circuit using a PIN diode in order to detune the coil during RF transmission. For each element, a housing was 3D printed to robustly attach the coaxial cable to the coil element, and also to prevent these cables from coiling.

The elements were arranged in phased arrays of three coil elements each (Fig. 1b) and two of such phased arrays were placed next to each other in such a way that the coupling of the elements was minimal. The initial tuning and matching values were calculated from theory, and the optimal values were subsequently determined by trial-and-error while loaded with a male volunteer. Although tuneable capacitors would ease the tuning and matching process, fixed capacitors were used for their lower losses.

The arrays were placed in a synthetic leather sheet. The 3D-printed housing and the synthetic leather insulated the patient from the coil and improved the robustness of the coil, while maintaining flexibility. The coil was fixed on the head by a Velcro strap around the chin and two flaps, which were placed between the headphones and the subject’s head (Fig. 1c).

### Quantitative assessment of coil performance

To quantitatively compare the flexible to the conventional coil, a 2D gradient echo sequence was used to acquire images for all five volunteers. The images were also reconstructed for each coil element separately. The imaging parameters were: TR/TE: 6.3/3.2 ms; voxel size: 1.25 × 1.25 × 1.25 mm^3^; flip angle: 8°; no parallel imaging; scan time: 52 s. To obtain noise-only images, this sequence was repeated with the gradients and RF transmitter switched off and the acquired noise was fed into the same reconstruction pipeline. In order to obtain g-factor maps, we repeated the sequence another five times for the last volunteer only with increasing SENSE acceleration factor along the left–right direction (1.2, 1.5, 2, 3, and 4). The pre-scans were not repeated between these acquisitions.

In MATLAB (R2018a, MathWorks, Natick, MA), we calculated the noise correlation matrix from data of a single volunteer, in which noise-only images are correlated between all possible combinations of coil elements [14]. To obtain the mean noise correlation of this matrix, we averaged the non-diagonal elements of this noise correlation matrix.

SNR maps were calculated by dividing the signal in a voxel from the actual image by the noise from the noise-only image, which was calculated as the standard deviation within a radius of three voxels surrounding that voxel. The standard deviation of the noise was corrected for the Rician distribution of this noise by multiplication with a factor of 0.655 [15]. In 3D Slicer [16], we manually segmented four regions: the masseter muscles, the parotid glands, and the anterior and posterior parts of the tongue, which were created by dividing the segmentation of the entire tongue into two roughly equally large parts using a coronal plane. For every region, the SNR gain was determined by dividing the average SNR of the flexible coil with the average SNR of the conventional coil. The average SNR gain was subsequently calculated by averaging over the five volunteers.

Using the vendor’s reconstruction software, we created g-factor maps for various acceleration factors for a single volunteer. To calculate the average g-factor in the head, we created a mask by applying a threshold on the magnitude images and subsequently smoothening the mask with morphological operations. By combining the regions of the anterior and posterior parts of the tongue, which were also used for the SNR gain calculation, we determined the average g-factor in the tongue.

### Diffusion-tensor multiband imaging

For all five volunteers, diffusion-tensor imaging (DTI) of the tongue was accelerated using a SE-EPI sequence with multiband (MB) SENSE [4]. For MB factor 1 (no MB acceleration), the imaging parameters were TR/TE: 2622/67 ms; scan time: 23.6 s. For MB factor 2, these imaging parameters were changed to TR/TE: 1405/71 ms; scan time: 12.6 s. Other imaging parameters that were the same for both acquisitions were: ETL: 35; matrix size: 64 × 64; voxel size: 3 × 3 × 3 mm^3^; in-slice SENSE factor: 1.5; no partial Fourier imaging; NSA: 1; SPIR and SSGR fat suppression; b-value: 700 s/mm^2^ along 6 different directions. An region-of-interest was manually drawn in the genioglossus muscle, in which the average apparent diffusion coefficient (ADC) and fractional anisotropy (FA) were calculated using MRtrix3 [17].

### Real-time MRI of swallowing

Single-slice midsagittal MRI scans of swallowing were acquired using a single-slice golden angle radial GRE sequence [18] with the following parameters: TR/TE: 2.9/1.12 ms; flip angle: 10°; TFE factor: 22; matrix size: 128 × 128; voxel size: 2 × 2 × 6 mm^3^; golden angle: 111.25°; scan time: 10 s. Volunteers were asked to swallow 10 mL of pineapple juice, which is a natural T1 contrast agent due to the presence of manganese [19].

The images were reconstructed off-line using the Berkeley Advanced Reconstruction Toolbox (version 0.4.01) [20] and MATLAB. Because we assumed that the sensitivity maps were constant over time, we estimated these sensitivity maps using eSPIRIT [21] from low resolution images created from all spokes. Subsequently, we binned eight spokes in each frame (without using a sliding window approach), resulting in 43 frames per second. The images were reconstructed with compressed sensing [22] using the alternating direction method of multipliers (ADMM) algorithm for 50 iterations with a locally low-rank constraint in space [23] (with regularisation parameter $\lambda =0.005$ and block size 8) and a total variation constraint over time [18] (with $\lambda =0.01$). The regularisation parameters were determined empirically based on the images from the flexible coil. A median filter over time (with a length of 5 frames) was used to remove residual radial streaking artefacts [24], and a non-local means filter (with filtering parameter $h=0.5$, standard deviation of the Gaussian kernel $a=0.5$, search window radius of 6 voxels, and similarity window of 5 voxels) to further suppress noise [25].

### Image grading

The movies of the real-time MRI of swallowing were graded by four speech therapists, who had between seven and twenty years of experience in reviewing videofluoroscopic swallowing studies. These ten movies, five for each coil, were presented to the reviewers in a random order blinded to these reviewers. We assessed three aspects of the swallowing movies, namely the visualisation of swallowing, the presence of motion artefacts, and the overall quality of the movies. For the visualisation of swallowing, we composed five questions distributed over the oral and pharyngeal phases of normal swallowing [26]. These five questions were: ‘How well is the *labial seal* visualised?’; ‘How well is the *contact of the tongue and alveolar ridge* visualised?’; ‘How well is the *velopharyngeal closure* visualised?’; ‘How well is the *hyoid elevation* visualised?’; ‘How well is the *contraction of tongue base and pharyngeal wall* visualised?’. For the effect of motion artefacts on the image quality, we composed the following three questions: ‘To what extent does *radial streaking* affect the quality of the movie?’; ‘To what extent does *inhomogeneity of intensity* affect the quality of the movie?’; ‘To what extent does *motion blurring* affect the quality of the movie?’. For the overall image quality, we composed only one question: ‘How do you grade the *overall quality* of the movie?’. The reviewers answered each question using a five-point Likert scale, in which 1 represented the lowest image quality (or most artefacts) and 5 the highest image quality (or fewest artefacts).

### Statistical analysis

For the four regions (masseter muscles, parotid glands, and anterior and posterior parts of the tongue), the difference in average SNR between both coils over all five volunteers was tested using a paired *t*-test. For both coils separately, we tested the difference in average ADC and FA values between MB factor 1 and 2 over all five volunteers with a paired *t*-test. For every question individually, we modelled the grades provided by the speech therapists by a multilevel proportional-odds model using the ordinal package in R [27]. In this model, we included one fixed effect, the used coil (conventional or flexible), and two random effects, the subject and the speech therapist. Both random effects were modelled with random intercepts and random slopes with the fixed effect (the used coil). The *P*-values of the effect that the used coil had on the grades were calculated by likelihood-ratio tests of the full model compared to the model without the fixed effect (the coil used).

## Results

**Fig. 2:**
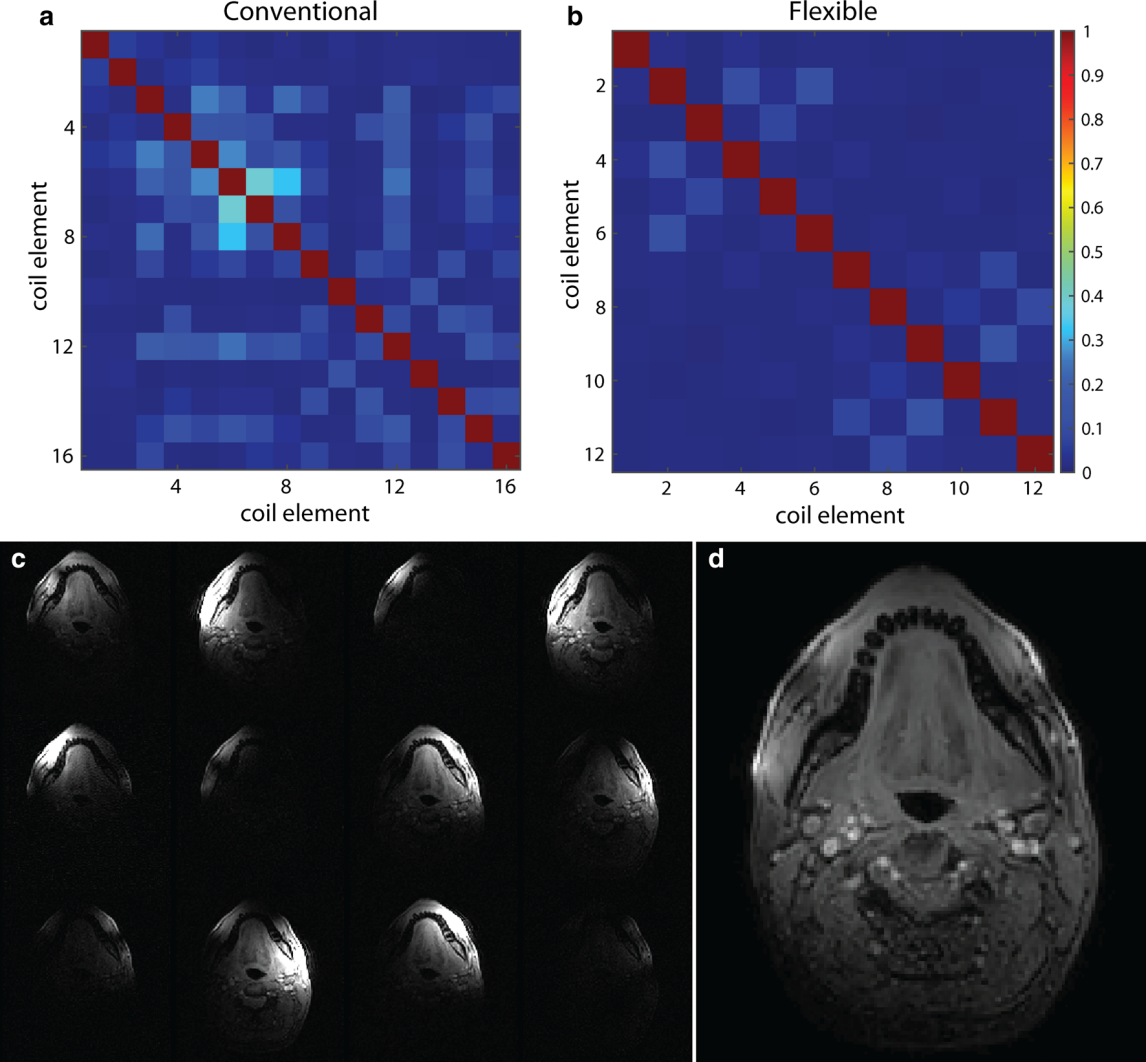
Signal and noise characteristics of the flexible coil: The noise correlation matrix of **a** the conventional and **b** the flexible coil for the first volunteer. For both coils, little coupling was present between coil elements, as the average non-diagonal elements of the noise correlation matrices was 2.3% for the flexible coil and 5.9% for the conventional coil. **c** The images of the same slice acquired by different coil elements confirmed that the small coil elements are sensitive to a small volume. A few images, such as the lower right-most image in (**c**), had a low average intensity because the corresponding coil element was located further from the imaged slice. **d** Combined magnitude image from all coil elements. Although the signal is generally fairly homogeneous, the image shows a few focal hyperintensities located close to the coil elements

The correlations in the noise-only images between all possible combinations of coil elements are visualised as a correlation matrix for the conventional coil (Fig. 2a) and the flexible coil (Fig. 2b). The mean noise correlation excluding the diagonal elements of the matrix was 5.9% for the conventional coil and 2.3% for the flexible coil. In the magnitude images of the coil elements (Fig. 2c), localised areas with high signal intensity can be appreciated, which would be beneficial to the parallel imaging performance of the coil. In this slice, the image intensity of four coil elements was lower, because these elements are sensitive to a region outside this slice. In the image of all elements combined (Fig. 2d), focal hyperintensities were present near the location of the coil elements.

**Fig. 3:**
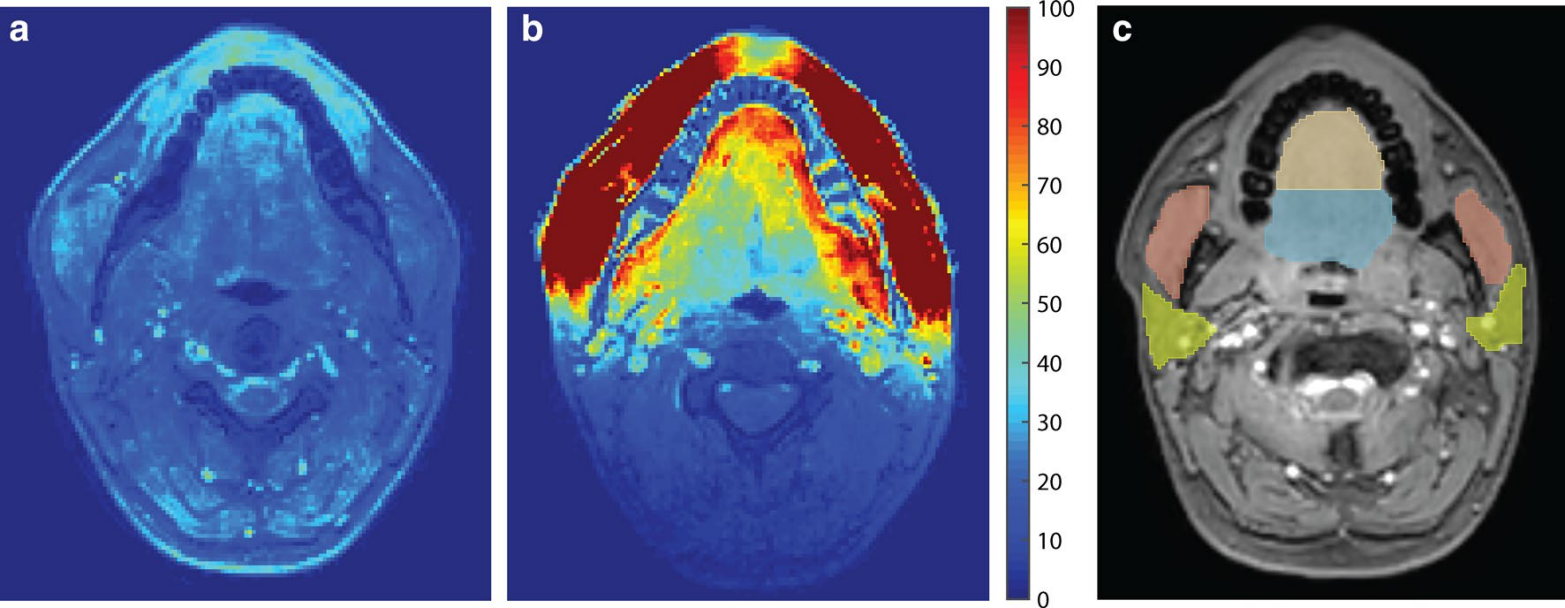
Signal-to-noise ratio evaluation. SNR maps of a similar slice acquired by **a** the conventional neurovascular coil and **b** the flexible tongue coil of one volunteer. The SNR is higher in the images from the flexible coil, especially in the cheeks. Also in our primary area of interest, the tongue, the SNR is higher. **c** The images were manually segmented into four regions, i.e. the anterior part of the tongue (brown), the posterior part of the tongue (blue), the masseter muscles (red), and the parotid glands (yellow)

In Fig. 3a and b the SNR maps are shown of both coils for a single volunteer. In the cheeks, where the coil elements are positioned, the SNR improvement over the conventional coil was the largest. Also in the tongue, the SNR was higher in the flexible coil. In Fig. 3c, a representative slice is presented containing the four regions that we manually segmented. Over all volunteers, the flexible coil array had a significant SNR gain in the following regions: an SNR gain of 2.1 in the anterior part of the tongue (*P* = 0.002), 2.0 in the posterior part of the tongue (*P* < 0.001) and 6.1 in the masseter muscles (*P* < 0.001). The SNR gain of 1.5 in the parotid glands was not significant (*P* = 0.171).

**Fig. 4:**
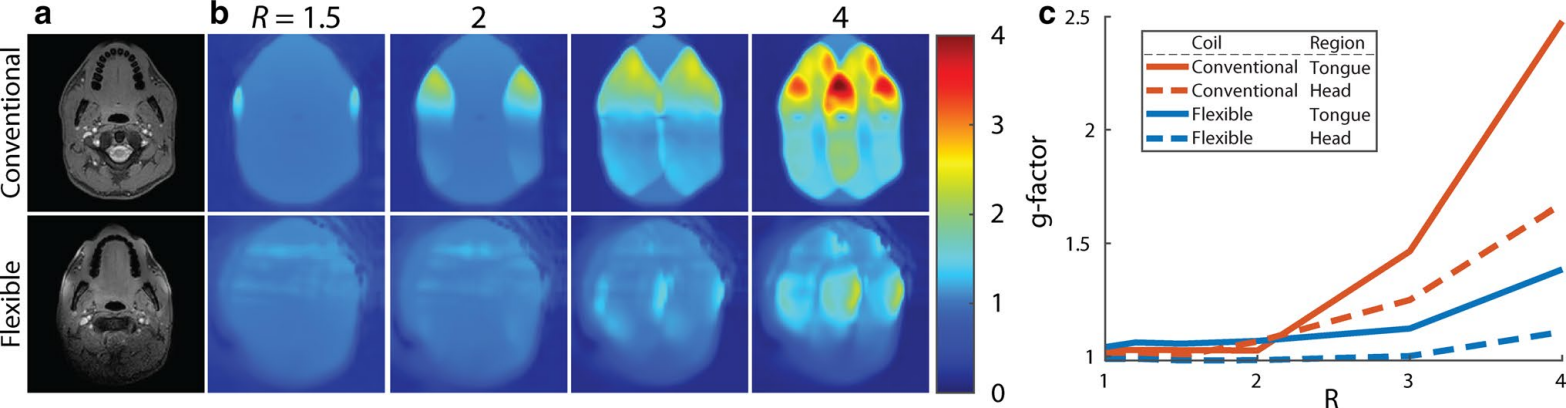
G-factor characteristics of the coil: **a** magnitude image without acceleration is given as anatomical reference. **b** For one volunteer, g-factor maps of the conventional coil and the flexible coil were created by increasing SENSE acceleration (*R*) in the left–right direction. The g-factor maps at *R* = 1 and *R* = 1.2 were not shown in this figure as these differed little from the g-factor map at *R* = 1.5. **c** Averaged g-factors over the head or the tongue are displayed for both coils. At low acceleration factors (*R* < 2), the conventional and flexible coils differed only slightly. At higher acceleration however, the g-factor increased more for the conventional coil than for the flexible coil

The g-factor evolution by increasing the SENSE acceleration is displayed in Fig. 4. Although the g-factor maps at low acceleration factors (*R* < 2) are similar, at higher acceleration factors (*R* ≥ 2), the g-factor increased more for the conventional coil than for the flexible coil. By averaging the g-factor over either the head or the tongue (Fig. 4c), a similar pattern could be appreciated, in which the g-factor increased more for the conventional coil.

**Fig. 5:**
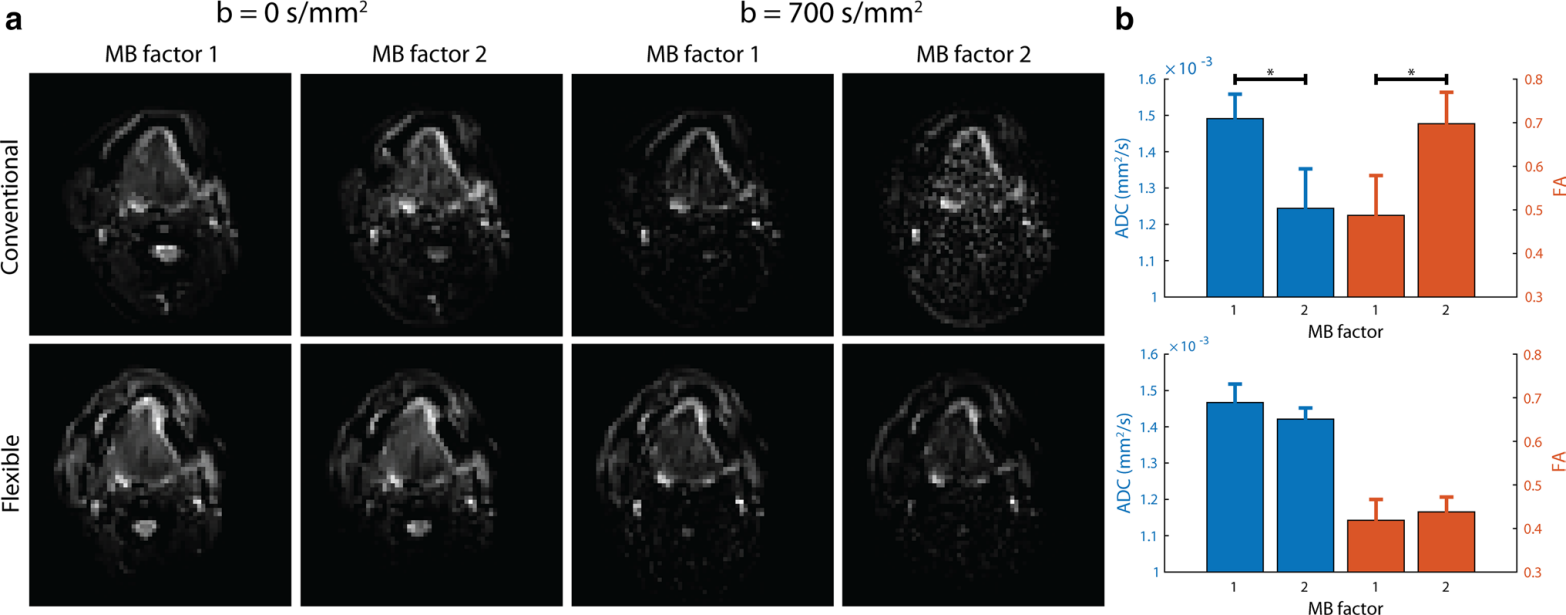
Diffusion-tensor imaging accelerated with multiband-SENSE. **a** For one volunteer, a similar slice is displayed for the conventional and flexible coil, with and without diffusion weighting, and using a MB factor of 1 or 2 (**a**). Although the noise amplification caused by MB-SENSE was low in the diffusion-weighted images from the flexible coil, this noise obscured the diffusion-weighted images acquired with the conventional coil. **b** The average and standard deviation of two DTI metrics, ADC and FA over the five volunteers were calculated within a region-of-interest in the genioglossus muscle. For the conventional coil, significant differences were found between MB factor 1 and 2 for both the ADC (*P* = 0.001) and the FA (*P* = 0.008). No such significant differences were found for the flexible coil

In Fig. 5, we show that diffusion-weighted imaging could be accelerated with MB-SENSE (MB factor 2) without unfolding artefacts. Without diffusion weighting, images accelerated with MB-SENSE appeared to be similar to images without MB-SENSE for both coils. With diffusion weighting however, the noise obscured the accelerated images from the conventional coil, while the accelerated images from the flexible coil were still similar to the non-accelerated images. For the flexible coil, no statistically significant differences were found between MB factor 1 and two for ADC (*P* = 0.196) and FA (*P* = 0.419). For the conventional coil however, using MB acceleration significantly changed both ADC (*P* = 0.001) and FA (*P* = 0.008).

**Fig. 6:**
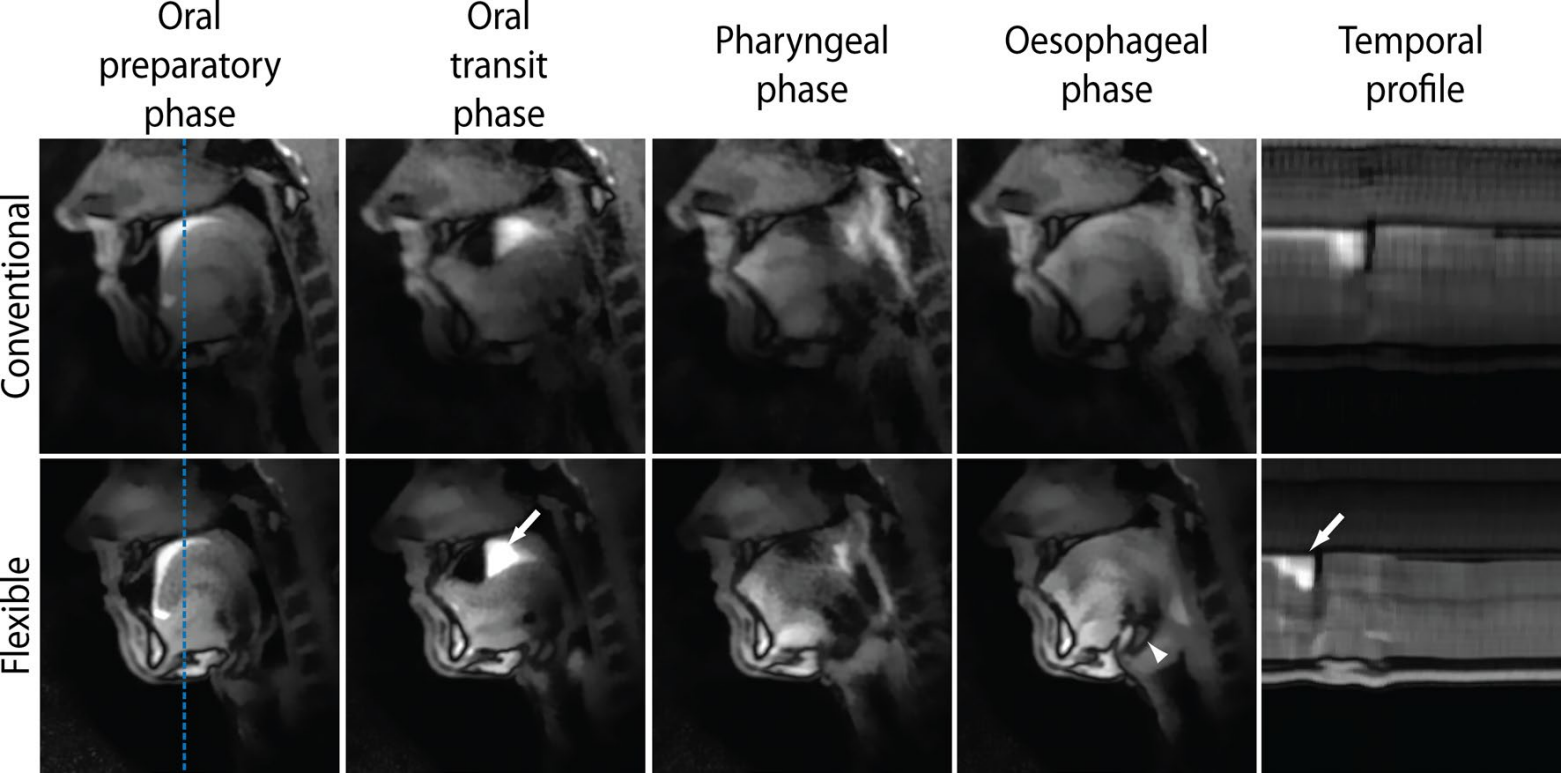
For the conventional and flexible coil, real-time MRI of swallowing at 43 frames per second is displayed for one volunteer. Four swallowing phases were distinguished: the oral preparatory phase, the oral transit phase, the pharyngeal phase, and the oesophageal phase. In the rightmost panels, the temporal profile of a single line, indicated by the blue dashed line, is shown. The white arrows, in the panels of the oral transit phase and the temporal profile for the flexible coil, indicate the swallowing contrast agent (pineapple juice). The white arrowhead, in the panel for the flexible coil in the oesophageal phase, indicates the hyoid, which could be significantly better visualised with the flexible coil (*P* = 0.029). The full movies of this volunteer are available for the conventional coil (Online Resource 1) and the flexible coil (Online Resource 2)

For the flexible and conventional coils, four frames from the real-time MRI of swallowing are displayed in Fig. 6. In these frames, four swallowing phases could be distinguished for both coils. The epiglottis and hyoid appeared to be visualised more sharply for the flexible coil. In the temporal profile, the contrast agent also appears to be visualised more sharply with the flexible coil. The differences between the conventional coil (Online Resource 1) and the flexible coil (Online Resource 2) may be better appreciated in the full movies of the real-time MRI of swallowing.

**Fig. 7:**
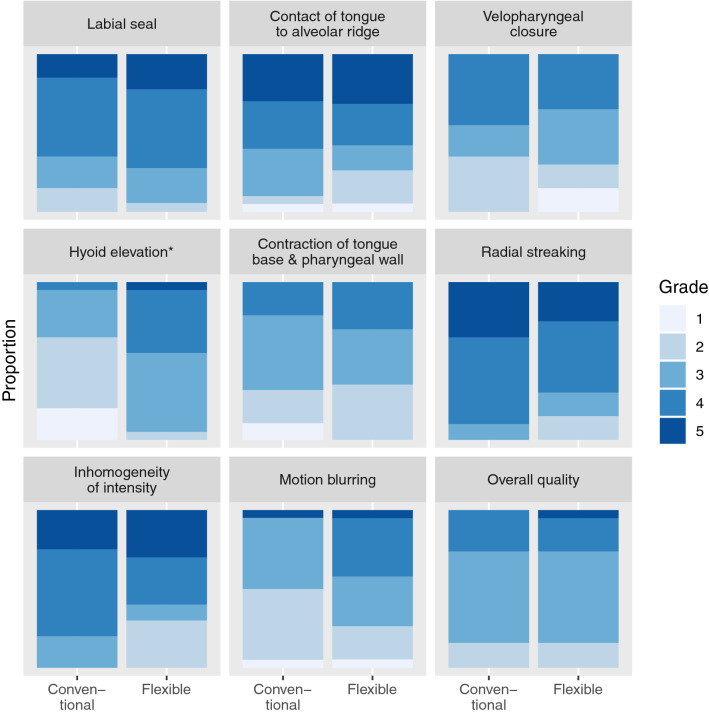
The proportions of grades given by the reviewers for the nine questions about the real-time MRI of swallowing. For this figure only, the grades for the five different subjects and by the four reviewers have been pooled. The grade 5 related to the best image quality or least artefacts, while 1 related to the worst image quality or most artefacts. For the visualisation of hyoid elevation, the odds of receiving a better grade was significantly higher for the flexible coil (*P* = 0.029) than for the conventional coil. For the other aspects of real-time MRI of swallowing, no differences in odds were found

The proportion of grades given by the speech therapists for either the conventional coil or the flexible coil are presented in Fig. 7. For all questions asked except for the question about hyoid elevation, there were no statistically significant differences in odds between the coils. For the visualisation of the hyoid elevation, the odds of being graded better were significantly higher for the flexible coil (*P* = 0.029).

## Discussion

In this study, we designed a flexible receiver coil for tongue MRI at 3T and evaluated its performance. Compared to a conventional coil, our flexible coil exhibits higher SNR in the tongue and lower g-factor values at high acceleration factors. For DTI of the tongue, the flexible coil facilitates faster scanning using MB-SENSE. For real-time MRI of swallowing using compressed sensing, similar image quality was found for both coils, except for the visualisation of hyoid elevation where the flexible coil performed better.

Although we found higher SNR values in the tongue with the flexible coil, the SNR rapidly decreases further away from the coil. In the parotid glands, the mean SNR no longer differed significantly between the two coils. In the back of the neck, the SNR appeared to be even lower for the flexible coil. Thus, imaging with the flexible coil is restricted to a smaller field-of-view than with the conventional coil. However, this field-of-view can easily be extended by using additional coil elements from a torso coil or the coils in the patient bed depending on the application. Another option would be to redesign the flexible coil with a higher number of coil elements. However, care should to taken to maintain the flexibility of the coil.

The flexibility of the presented tongue coil leads to the following three advantages: The coil should be more comfortable for the subject, the coil elements are closer to the volume of interest, which increases SNR, and the flexibility of the coil allows us to use auxiliary equipment during scanning, which means that we could easily administer the swallowing contrast agent. The access to auxiliary equipment also facilitates the implementation of other study protocols, such as concurrent manometry and MRI for patients with dysphagia [8], or the evaluation of obstructive sleep apnoea with MRI while patients are wearing a facial mask [28].

However, the flexibility also allows the subject to move more during the scans, which may result in more motion artefacts than with conventional coils. To prevent head rotation during scans, we therefore made a 3D printed headrest. More subtle motion originating from breathing and swallowing may still result in motion artefacts, but these artefacts are expected to occur less often due to acceleration with techniques such as MB imaging.

Other custom receiver coils for tongue or upper airway imaging have been reported for both 1.5T [10] and 3T [13]. Although these coils appear to be more rigid than the flexible coil presented in this paper, their reported SNR gains are higher, i.e. SNR gains in the tongue of between 2.6 and 5.5 for the coil at 3T. These differences in SNR gain may be explained by the different conventional coils used as a reference (an 8-channel coil compared to a 16-channel coil in this study).

Using the flexible coil, we were the first to show the feasibility of MB acceleration in the head and neck area, as we were able to accelerate DTI by a MB factor of 2, without significantly affecting the ADC or FA. With the conventional coil however, the ADC was significantly lower and the FA was significantly higher between MB factor 1 and 2, which is consistent with the effect that a low SNR level has on these metrics [29]. Using MB imaging, we can now reduce the scan time high-angular resolution diffusion-weighted imaging of the tongue [9], which would otherwise require too much scan time in a clinical setting. Other sequences that rely on an EPI read-out such as arterial spin labelling in the parotid glands [30], could also benefit from MB imaging. Multiband imaging can even be combined with a radial acquisition [31], which we used for the real-time MRI of swallowing.

We showed that the SNR gain from the flexible coil only modestly improves the image quality of real-time imaging of swallowing. Although we measured a two-times higher SNR and a lower g-factor at high acceleration factors for the flexible coil, no difference in image quality was found in the real-time MRI of swallowing, except for hyoid elevation. For the flexible coil, the temporal profile was less blurred than for the conventional coil, but no significant decrease in image quality due to motion blurring was detected. As the reviewers had not graded real-time MRI of swallowing before, we might have detected smaller differences in image quality by training the reviewers more extensively. Nevertheless, a radial acquisition in combination with compressed sensing reconstruction is apparently effective in restoring image quality in undersampled data. To benefit more from the improved SNR of the flexible coil, we believe that the in-plane resolution should be increased or that multiple slices should be acquired. The latter is especially useful in cases where aspiration may be missed, if it is located laterally from a single midsagittal slice.

Real-time MRI of swallowing was previously demonstrated to be feasible for a frame rate of 24.3 frames per second [19]. Although we attained a higher frame rate, these previous studies were able to reconstruct the movies in real time using the regularised non-linear inversion method [32]. The advantage of such a real-time reconstruction method is that images can be made available for the examiner at the scanner immediately. In this way, the acquisition may be adjusted or repeated as necessary, which may help the integration of real-time MRI of swallowing into clinical practice.

The main limitation of the flexible coil are the focal hyperintensities near the coil elements. These are caused by the signal profile of the small receive elements that has a steep signal drop-off the further you move from the element. Although these hyperintensities did not affect the image quality in the sagittal images of swallowing graded by speech therapists, they were more clearly present in transverse images. These inhomogeneities were also reported in other flexible receiver coils [33]. We tested several correction methods, such as normalisation using the coil sensitivity maps, but we could not find a method that sufficiently homogenised the images. Until such a method has been developed, it is possible to reduce the inhomogeneity by placing the coil slightly further from the skin, but this will reduce the SNR.

In conclusion, the 12-channel flexible coil provides a higher average SNR and lower g-factor in the tongue, which enable higher image acceleration than a conventional neurovascular coil. This acceleration may not only reduce the incidence of motion artefacts, but also allows the application highly-accelerated MRI protocols such as multiband diffusion tensor imaging and real-time MRI of swallowing at 43 frames per second.

## Electronic supplementary material

Below is the link to the electronic supplementary material.

Supplementary file1 A movie of real-time MRI of swallowing at 43 frames per second acquired with the conventional coil. Pineapple juice was used as a contrast agent. Flickering is clearly noticeable in this movie (WMV 1328 kb)

Supplementary file2 A movie of real-time MRI of swallowing at 43 frames per second acquired with the flexible coil. Pineapple juice was used as a contrast agent. Flickering is not as noticeable in this movie. However, the signal intensity is more inhomogeneous across the image (WMV 1053 kb)
